# The Effects of Inorganic Phosphorus Levels on Phosphorus Utilization, Local Bone-Derived Regulators, and BMP/MAPK Pathway in Primary Cultured Osteoblasts of Broiler Chicks

**DOI:** 10.3389/fvets.2022.855405

**Published:** 2022-03-22

**Authors:** Tingting Li, Sumei Cao, Xiudong Liao, Yuxin Shao, Liyang Zhang, Lin Lu, Zongping Liu, Xugang Luo

**Affiliations:** ^1^Poultry Mineral Nutrition Laboratory, College of Animal Science and Technology, Yangzhou University, Yangzhou, China; ^2^Mineral Nutrition Research Division, State Key Laboratory of Animal Nutrition, Institute of Animal Science, Chinese Academy of Agricultural Sciences, Beijing, China; ^3^College of Veterinary Medicine, Yangzhou University, Yangzhou, China

**Keywords:** BMP/MAPK pathway, broiler, local bone-derived regulator, phosphorus utilization, tibial osteoblast

## Abstract

Understanding the underlying mechanisms that regulate the bone phosphorus (P) utilization would be helpful for developing feasible strategies to improve utilization efficiency of P in poultry. We aimed to investigate the effects of inorganic P levels on P utilization, local bone-derived regulators and bone morphogenetic protein/mitogen-activated protein kinase (BMP/MAPK) pathway in primary cultured osteoblasts of broiler chicks in order to address whether local bone-derived regulators or BMP/MAPK pathway was involved in regulating the bone P utilization of broilers using an *in vitro* model. The primary cultured tibial osteoblasts of broiler chicks were randomly divided into one of five treatments with six replicates for each treatment. Then, cells were respectively incubated with 0.0, 0.5, 1.0, 1.5, or 2.0 mmol/L of added P as NaH_2_PO_4_ for 24 days. The results showed that as added P levels increased, tibial osteoblastic P retention rate, number and area of mineralized nodules, the mRNA expressions of endopeptidases on the X chromosome (*PHEX*), dentin matrix protein 1 (*DMP1*), bone morphogenetic protein 2 (*BMP2*), and the mRNA and protein expressions of matrix extracellular phosphoglycoprotein (MEPE) increased linearly (*p* < 0.001) or quadratically (*p* < 0.04), while extracellular signal-regulated kinase 1 (*ERK1*) mRNA expression and c-Jun N-terminal kinase 1 (JNK1) phosphorylated level decreased linearly (*p* < 0.02) or quadratically (*p* < 0.01). Correlation analyses showed that tibial osteoblastic P retention rate was positively correlated (r = 0.452–0.564, *p* < 0.03) with *MEPE* and *BMP2* mRNA expressions. Furthermore, both number and area of mineralized nodules were positively correlated (r = 0.414–0.612, *p* < 0.03) with *PHEX, DMP1, MEPE*, and *BMP2* mRNA expressions but negatively correlated (r = −0.566 to −0.414, *p* < 0.04) with the *ERK1* mRNA expression and JNK1 phosphorylated level. These results suggested that P utilization in primary cultured tibial osteoblasts of broiler chicks might be partly regulated by PHEX, DMP1, MEPE, BMP2, ERK1, and JNK1.

## Introduction

Phosphorus (P) is an essential macro-mineral required for bone development, growth, and productivity (1–3) and also the third most expensive component in poultry diets (4). Dietary P deficiency can cause a reduced feed intake, growth retardation, and other skeletal deformities of broilers (5–8). Therefore, sufficient P supply in their diets is vital to meet metabolic requirements. On the other hand, excessive P over requirements is excreted through manure, leading to both economical loss and environmental pollution (9, 10). Concerns about the environmental burden caused by high P excretion and the existing status of scarce P resources make it imperative to strive for more efficient P utilization of broilers.

Approximately 80% of P absorbed from the small intestine is deposited in the bone as hydroxyapatite (11), and this deposition process is mainly carried out by osteoblasts (12). Furthermore, the uptake of P by osteoblasts is usually considered as a major factor regulating the bone development, mineralization, and remodeling (13). Therefore, primary cultured osteoblasts may be an ideal *in vitro* model for exploring the patterns of bone P utilization in broilers. Possible traits related to bone P utilization *in vitro* are tibial osteoblastic P retention rate and number and area of mineralized nodules; among them, the number and area of mineralized nodules are often used as response indicators in *in vitro* studies of calcium/P deposition (14–16). Local bone-derived regulators, such as fibroblastic growth factor 23 (FGF23), matrix extracellular phosphoglycoprotein (MEPE) and dentin matrix protein 1 (DMP1), and phosphate-regulating gene with homologies to endopeptidases on the X chromosome (PHEX) have been found to be involved in regulating P homeostasis and bone mineralization (17, 18). One latest study from our laboratory suggested that bone development and P retention might be partly regulated by FGF23, DMP1, and PHEX (5), implying possible contributions of local bone-derived regulators to bone P utilization of broilers. In addition to local bone-derived regulators, multiple signaling pathways are essential components during osteoblast mineralization, such as bone morphogenetic protein/mitogen-activated protein kinase (BMP/MAPK) pathway (19). As a member of the BMP family, BMP2 is a potent inducer governing the osteoblastic mineralization (20, 21). Furthermore, accumulating evidence have revealed that BMP2 exerts its osteogenic function *via* activating MAPK signaling pathway (22, 23). Another latest study from our laboratory further suggested that BMP/MAPK pathway might play a potential regulatory role in bone development and P retention of broilers (24). However, the results from the above latest studies *in vivo* with broilers might be affected by other factors like the feed intake change except for P, and the specific and direct effect of P itself on the above aspects of broilers is unknown. Therefore, it is necessary to investigate the specific and direct effect of P itself on the above aspects using the primary cultured model of osteoblasts of broiler chicks. According to the results of our above latest studies *in vivo*, it is reasonable to hypothesize that P utilization in an osteoblastic model *in vitro* might be partly regulated by local bone-derived regulators and BMP/MAPK pathway. Therefore, the objective of the current study was to investigate the effects of added inorganic P levels on P utilization parameters, local bone-derived regulators, and BMP/MAPK pathway in primary cultured tibial osteoblasts of broiler chicks to test the above hypothesis.

## Materials and Methods

### Isolation and Cultivation of Primary Tibial Osteoblasts of Broiler Chicks

The primary tibial osteoblasts of broiler chicks were isolated and cultivated by using a modified approach of the previous method reported in mouse (25). Tibias from both legs were obtained from 1-day-old commercial chicks (male Arbor Acres broilers, purchased from Huadu Broiler Breeding Corp., Luanping, China) after cervical dislocation. The birds were soaked in alcohol for 2 min after cervical dislocation. Legs were removed from the hip joint and metacarpal. Dissected legs were kept in Dulbecco's modified Eagle's medium (DMEM; Solarbio, Beijing, China) containing 1% penicillin-streptomycin (Gibco, Grand Island, NY, USA) until the connective tissues and muscles were completely removed. The epiphysis of the tibia was removed to expose the bone marrow cavity. Bone marrow inside the bone was flushed with washing buffer in a syringe. The tibias were split longitudinally with a scalpel and then scraped with a curved forceps to remove the hematopoietic cells adhered to the compact bones. The clean bone flakes were cut into 1 mm^2^ with sterile surgical scissors, plated in 60-mm cell culture dishes, and then incubated in the complete culture medium, consisting of DMEM, supplemented with 1% penicillin-streptomycin, 1% L-glutamine (Gibco, Grand Island, NY, USA), and 15% fetal bovine serum (FBS; Gibco, Auckland, New Zealand) at 37°C in a humidified atmosphere containing 5% CO_2_. Once the cells migrated from bone pieces and covered up to 80% of the flask bottom surface, the cells were washed twice with Dulbecco's phosphate-buffered saline (D-PBS) without CaCl_2_ and MgCl_2_ (Gibco, Grand Island, NY, USA), dissociated with 0.25% Trypsin-EDTA (Solarbio, Beijing, China) for 1 min, and subcultured in six-well plates at a density of 5 × 10^5^ cells/well in the above complete culture medium. This passage was marked as P1. The P1 cells were identified by alkaline phosphatase (ALP), immunofluorescence, and alizarin res S (ARS) staining (26), and then used in the following experiments.

### Experimental Design and Treatments

A one-factor completely randomized design with six replicates was adopted in the present study. Once the P1 osteoblasts grew to ~80–90% confluence, the cells were washed with the P-free medium (DMEM with no phosphate, Gibco, Grand Island, NY, USA), randomly divided into one of five treatments, and then incubated in the P-free medium supplemented with both 15% FBS and 0.0, 0.5, 1.0, 1.5, or 2.0 mmol/L of P as NaH_2_PO_4_, respectively, for 24 days. The total P levels in the above media were 0.568, 1.055, 1.513, 2.010, and 2.562 mmol/L by analysis, respectively, and calcium level in each medium used in the present study was 2.2 mmol/L by analysis. The reason for selecting NaH_2_PO_4_ as the P source is that the original P source in the osteoblastic DMEM culture is NaH_2_PO_4_, while the P source (CaHPO_4_) usually used in the diet of broilers is less soluble in DMEM. The supplemented P levels were based on the study of Zoidis et al. (27). The medium was replaced every 2 days. The optimal incubation time (24 days) was chosen based on the results of our latest study (28).

### Bone ALP Staining

When the P1 cells reached 100% confluence, cells were fixed with 4% formaldehyde (Solarbio, Beijing, China) for 10 min and then washed in PBS three times for 5 min each. Subsequently, the fixed cells were stained by the ALP strain kit (Beyotime, Shanghai, China). The stained cells were observed and photographed by an optical microscope (Nikon, Japan). The cells strained in purple blue were osteoblasts.

### Immunofluorescence

When the P1 cells reached 100% confluence, cells were fixed with 4% formaldehyde for 30 min and then washed in PBS three times for 5 min each. The fixed cells were then permeabilized with 0.2% Triton X-100 (Solarbio, Beijing, China) for 15 min and then blocked with normal goat serum (Solarbio, Beijing, China) for 30 min at 37°C. After blocking, the cells were incubated with a primary antibody against collagen type I alpha 1 (COL1A1; Boster, Wuhan, China) overnight at 4°C. Afterwards, the cells were then incubated with a DyLight-488 labeled goat-anti-rabbit IgG secondary antibody (Boster, Wuhan, China) for 1 h at 37°C. The cell nuclei were visualized using 4′,6-diamidino-2-phenylindole (DAPI) staining solution (Beyotime, Shanghai, China). Finally, images were captured with a fluorescence microscope (Leica, Wetzlar, Germany) by randomly selecting five fields. The COL1A1-positive cells showed green fluorescence. As for the cell purity, the percentage of COL1A1-positive cells was calculated as (number of COL1A1-positive cells)/(total number of cells in the field) × 100%.

### ARS Staining

The mineralized module formation was detected by ARS straining as previously described (29). When cultured for 24 days, the cells were washed with PBS and fixed in 4% formaldehyde for 15 min at room temperature. The cells were then washed twice with D-PBS prior to addition of 1 ml of 1% ARS (pH 4.2) per well. The plates were incubated at room temperature for 20 min with gentle shaking, followed by thrice washing with D-PBS. Mineralized nodules were positive to ARS.

### Tibial Osteoblastic P Retention Rate

*In vivo*, tibia P retention rate was used to evaluate the bone P utilization of broilers (30); therefore, tibial osteoblastic P retention rate was determined *in vitro* to evaluate the osteoblastic P utilization. To determine the tibial osteoblastic P retention rate, the old medium was collected and pooled at every medium replacement. The total P contents in the fresh or old medium were determined by inductively coupled plasma optical emission spectrometry [5110 ICP-OES, Agilent Technologies Australia (M) Pty Ltd, Australia]. Accurately 1.0 ml of the fresh or old medium was taken in triplicate and digested with 12.5 ml of HNO_3_ and 2.5 ml of HClO_4_ at 200°C in a 50-ml calibrated flask until the solution became clear, and it was evaporated to almost dryness and diluted to about 15 ml with 2% HNO_3_ before analyses. Validation of the mineral analysis was conducted using soybean meal (GBW10013; National Institute of Standards and Technology, Beijing, China) as a standard reference material. The tibial osteoblastic P retention rate was calculated as follows:


$$\begin{array}{l} \text{Tibial osteoblastic}\\ \text{P retention rate }\left(\text{\%}\right)=\;\frac{\text{V}1\;\times \text{ C}1-\text{V}2\;\times \text{ C}2}{\text{V}1\;\times \text{ C}1}\times \;100, \end{array}$$

where V1 is total volume (ml) of the added fresh medium, C1 is total P content (mmol/L) in the fresh medium, V2 is total volume (ml) of the pooled old medium, and C2 is total P content (mmol/L) in the old medium.

### Mineralized Nodule Formation in Tibial Osteoblasts

The mineralized nodule formation was detected by ARS staining as described in ARS Staining. After staining, the number of positively stained mineral nodules was measured by visual counting under an optical microscope. Furthermore, the area of the mineralized nodules in five randomly selected visual fileds of each replicate well was analyzed with Image Pro-Plus 6.0 software (Media Cybernetics, USA).

### Quantitative Real-Time Polymerase Chain Reaction

After 24 d treatments with various P concentrations, the mRNA expression levels of local bone-derived regulator genes (*FGF23, MEPE, DMP1* and *PHEX*) and BMP/MAPK pathway-related genes [BMP2, extracellular signal-regulated kinase 1 (ERK1), c-Jun N-terminal kinase 1 (JNK1) and p38 mitogen-activated protein kinase alpha (p38α)] were evaluated by real-time quantitative polymerase chain reaction (RT-qPCR) as described by Li et al. (31). Briefly, according to the manufacturer's instruction, PrimeScriptTM RT Master Mix kit (TaKaRa, Dalian, China) was used to reverse RNA into cDNA. Subsequently, 50 ng of total cDNA was applied for RT-qPCR analysis using SYBR-Green PCR Master Mix (Life Technologies, Carlsbad, CA, USA). Finally, the relative expression of the target genes was calculated using the 2^−ΔΔCt^ method, and the data were normalized by the geometric mean of two internal reference genes (β-actin and *GAPDH*). All primer sequences are presented in Table 1.

**Table 1:** Primers used for the target and reference genes.

**Genes**	**Sequence (5^**′**^-3^**′**^)**	**Product length (bp)**	**GenBank ID**
*FGF23*	Forward: ATGCTGCTTGTGCTCTGTATC	189	XM_425663.4
	Reverse: ACTGTAAATGGTTTGGTGAGG		
*MEPE*	Forward: ACCTCAACCAGTGTGGAAGG	114	NM_204569.1
	Reverse: ATCTCCATGAGGTCCACCAG		
*DMP1*	Forward: GCCTGACGATGATGCTCCAA	116	NM_206993.1
	Reverse: TGGATGTGCTCTCTTCGCTC		
*PHEX*	Forward: TTTACGAGCCCAACTACTGT	194	NM_001397884.1
	Reverse: TTGTCATATTTCCGACCAT		
*BMP2*	Forward: GTTTGTGGTGGAGGTGGTTC	238	NM_204358.1
	Reverse: GTCCACATACAACGGATGCC		
*ERK1*	Forward: TCTTACTGCGCTTCAGGCAT	158	NM_204150.1
	Reverse: AATGTGGTCGTTGCTGAGGT		
*JNK1*	Forward: AGAGGGAGCACACAATAGAA	165	XM_040675398.1
	GAATG		
	Reverse: TTGACAGATGACGACGA		
	AGATGG		
*p38α*	Forward: ACGTGCAGTTCCTCATATACCA	145	XM_040691290.1
	Reverse: TGTCGAGCCAAGCCAAAATC		
β-actin	Forward: CAGCCATCTTTCTTGGGTAT	169	NM_205518.1
	Reverse: CTGTGATCTCCTTCTGCATCC		
*GAPDH*	Forward: GCACGCCATCACTATCTT	82	NM_204305.1
	Reverse: GGACTCCACAACATACTCAG		

*FGF23, fibroblast growth factor 23; MEPE, matrix extracellular phosphoglycoprotein; DMP1, dentin matrix protein 1; PHEX, phosphate-regulating gene homologous to endopeptidase on X chromosome; BMP2, bone morphogenetic protein 2; ERK1, extracellular signal-regulated kinase 1; JNK1, c-Jun N-terminal kinase 1; p38α, p38 mitogen-activated protein kinase alpha; GAPDH, glyceraldehyde-3-phosphate dehydrogenase*.

### Western Blotting

At the end of the P treatments, whole cell lysates were harvested in RIPA buffer (Beyotime, Shanghai, China). Cell lysates containing 30 μg of each protein were then subjected to a 10% sodium dodecyl sulfate–polyacrylamide gel electrophoresis (SDS-PAGE) and transferred onto a polyvinylidene difluoride membrane (Merck Millipore, Billerica, MA, USA). After blocking with 5% bovine serum albumin, the membrane was incubated overnight at 4°C with primary antibody against FGF23 (Abclonal, Wuhan, China), PHEX (Abclonal, Wuhan, China), DMP1 (Abcam, Cambridge, UK), MEPE (Abcam, Cambridge, UK), BMP2 (Abclonal, Wuhan, China), ERK1 (Abcam, Cambridge, UK), JNK1 (Abcam, Cambridge, UK), p38α (Abclonal, Wuhan, China), phosphorylated (p-)ERK (Abclonal, Wuhan, China), p-JNK1 (Abclonal, Wuhan, China), p- p38α (Abclonal, Wuhan, China), β-actin (Huaxingbio, Beijing, China), or β-tubulin (Huaxingbio, Beijing, China). After washing with Tris-buffered saline containing 0.02% (v/v) Tween-20 (TBST) three times for 5 min each, the membranes were developed with the secondary antibody of goat anti-rabbit (Huaxingbio, Beijing, China) or goat anti-mouse (Huaxingbio, Beijing, China). After washing with TBST three times for 5 min each, protein bands were visualized using enhanced chemiluminescence substrate (Tanon, Shanghai, China) and quantified with an ImageQuant LAS4000 scanner (GE Healthcare Life Sciences, Pittsburgh, PA), followed by analysis with TotalLab Quant Software (TotalLab, Newcastle on Tyne, UK). The β-actin or β-tubulin protein was used to normalize the expression levels of target proteins.

### Statistical Analyses

Data from the present study were analyzed by SAS statistical software (version 9.4, SAS Institute Inc., Cary, NC). The data were first tested for normality and homogeneity of variance using the Shapiro–Wilk and F-tests, respectively. Then, the data were subjected to one-way ANOVA using the general liner model (GLM) procedure, and the treatment comparisons for significant differences in all figures were tested by the least significant difference (LSD) method. Orthogonal polynomials were applied for testing linear and quadratic effects of dependent variables to independent variables. Simple correlation analyses between the P utilization-related parameters and local bone-derived regulators or BMP/MAPK pathway were carried out based on replicate values from different P level treatments using the Pearson procedure (5, 32). The replicate served as the experimental unit. The statistical significance was set at *p* ≤ 0.05.

## Results

### Morphological Features and Identification of Primary Cultured Tibial Osteoblasts of Broilers

**Figure 1:**
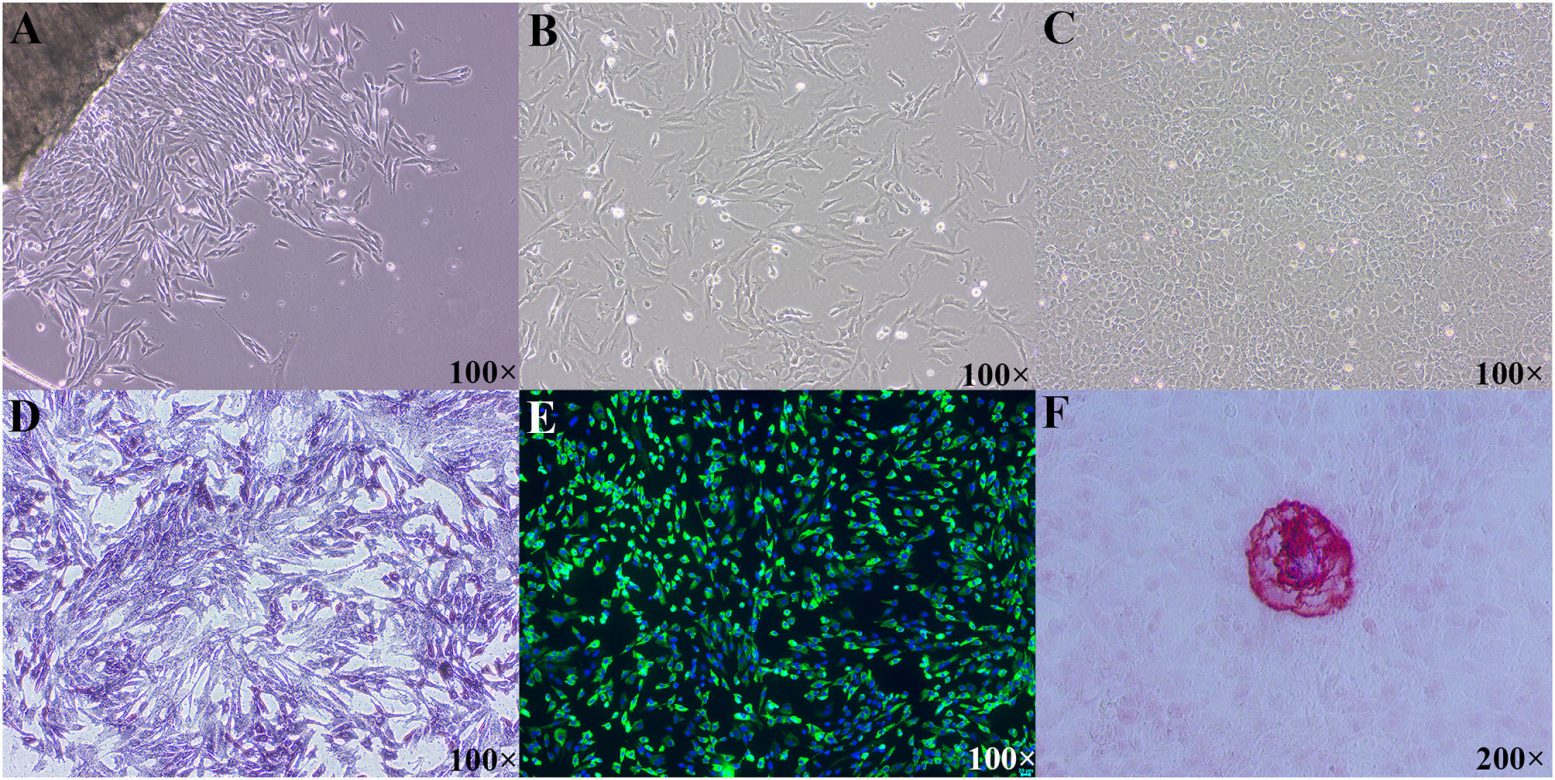
Morphological features and identification of primary cultured tibial osteoblasts of broiler chicks. **(A)** Morphological image of P0 cells, day of 3 culture; **(B)** morphological image of P1 cells, day of 2 culture; **(C)** morphological image of P1 cells, day of 10 culture; **(D)** primary osteoblasts were positive in ALP straining; **(E)** immunofluorescence of COL1A1 in the primary tibial osteoblasts; **(F)** mineralized nodules were positive in ARS staining. ALP, alkaline phosphatase; COL1A1, collagen type I alpha 1; ARS, alizarin res S.

As shown in Figures 1A,B, both P0 and P1 cells appeared typical morphological features of osteoblasts, such as multi-angle or fusiform shape. With the extension of culture time, cells grew like pave road stone (Figure 1C). Moreover, P1 cells showed the osteoblastic phenotypes, such as their synthesis of ALP (Figure 1D), collagen I (Figure 1E), and formation of mineralized nodules (Figure 1F). Immunofluorescence staining with osteoblast-specific antibody COL1A1 showed that the positive rate of osteoblasts was over 95%, indicating that the purity of the primary osteoblasts was high. Taken together, the cultured cells were osteoblasts with a high purity and can be used in the next experiment.

### Tibial Osteoblastic P Retention Rate and Number and Area of Mineralized Nodules

**Figure 2:**
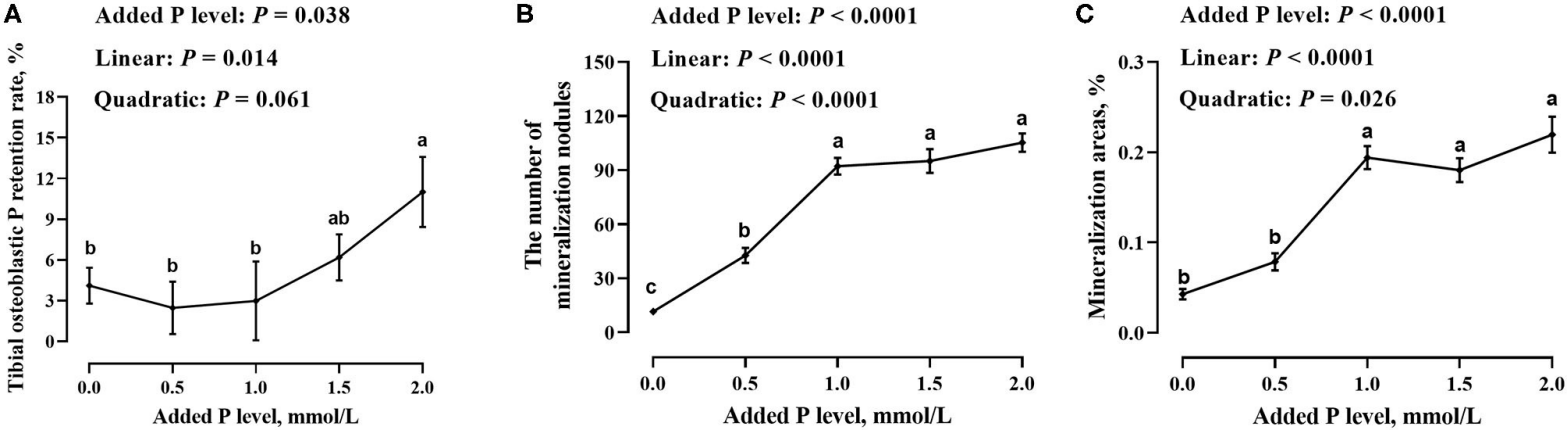
Effect of added P level on tibial osteoblastic P retention rate **(A)** and number **(B)** and area **(C)** of mineralized nodules in primary cultured tibial osteoblasts of broiler chicks. Each value was mean ± SE, *n* = 6. Means without a common letter differ, *p* ≤ 0.05.

The added P level had an effect (*p* ≤ 0.038) on the tibial osteoblastic P retention rate and number and area of mineralized nodules in primary cultured tibial osteoblasts of broiler chicks (Figure 2). The tibial osteoblastic P retention rate increased linearly (*p* = 0.014), whereas the number and area of mineralized nodules increased linearly (*p* < 0.0001) and quadratically (*p* ≤ 0.026) as added P levels increased and reached a stabilized level (*p* > 0.141) at 1.0–2.0 mmol/L of added P.

### MRNA and Protein Expressions of Local Bone-Derived Regulators

**Figure 3:**
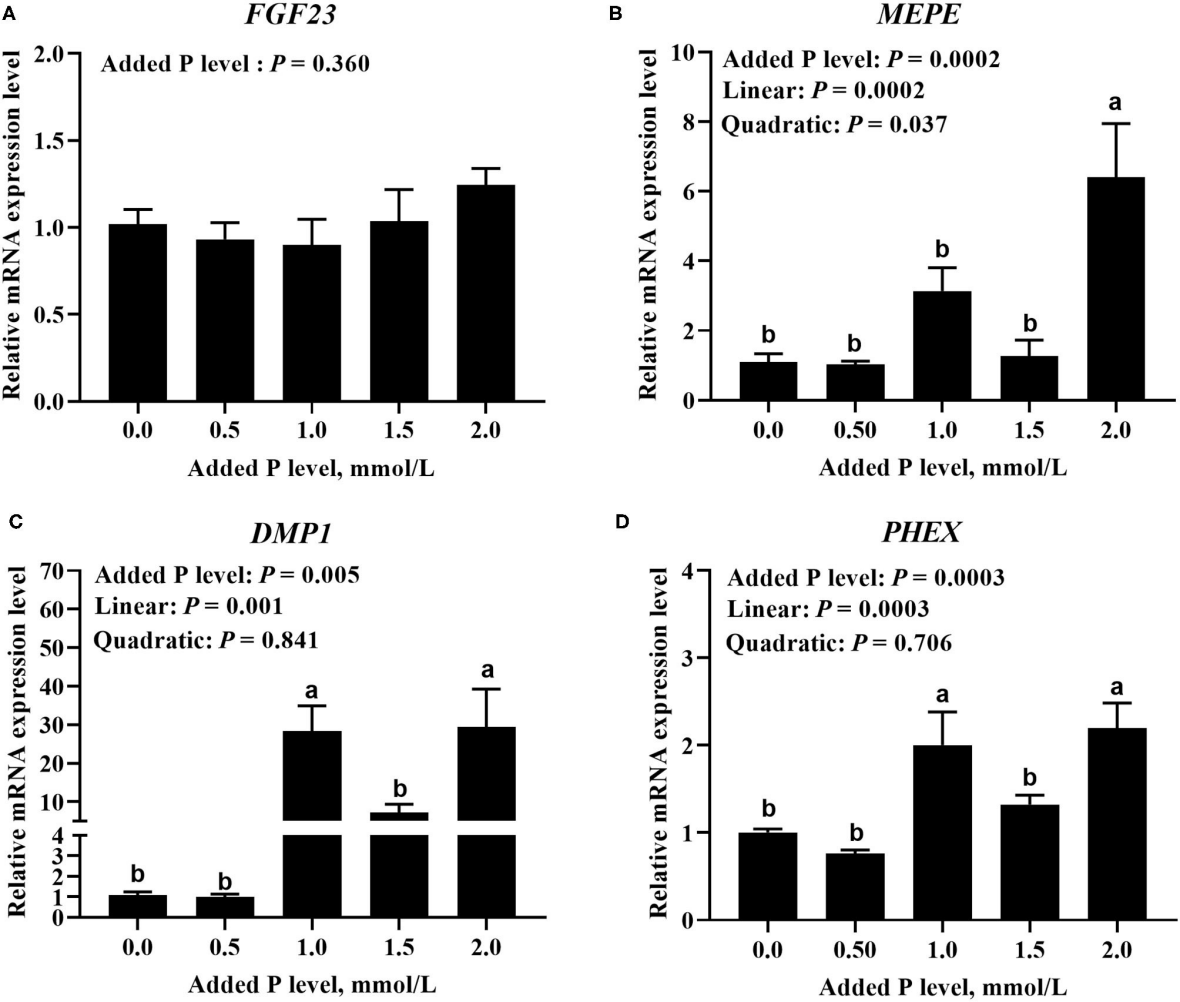
Effect of added P level on the mRNA expression levels of *FGF23* **(A)**, *MEPE* **(B)**, *DMP1* **(C)**, and *PHEX* **(D)** in primary cultured tibial osteoblasts of broiler chicks. Each value was mean ± SE, *n* = 6. Means without a common letter differ, *p* ≤ 0.05. *FGF23*, fibroblast growth factor 23; *MEPE*, matrix extracellular phosphoglycoprotein; *DMP1*, dentin matrix protein 1; *PHEX*, phosphate-regulating gene homologous to endopeptidase on X chromosome.

**Figure 4:**
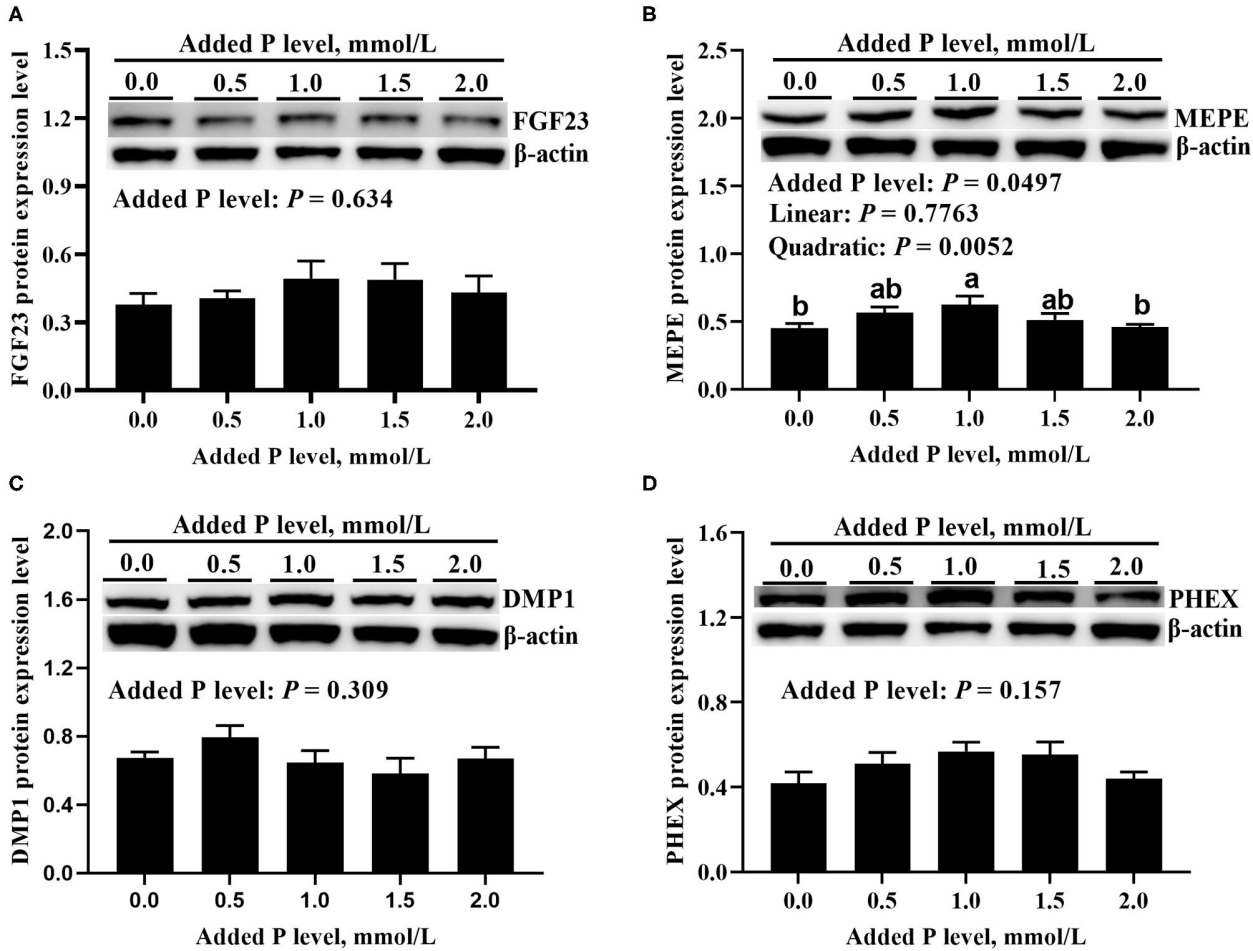
Effect of added P level on the protein expression levels of FGF23 **(A)**, MEPE **(B)**, DMP1 **(C)**, and PHEX **(D)** in primary cultured tibial osteoblasts of broiler chicks. Representative western blots for these proteins were shown. Each value was mean ± SE, *n* = 6. Means without a common letter differ, *P* ≤ 0.05. FGF23, fibroblast growth factor 23; MEPE, matrix extracellular phosphoglycoprotein; DMP1, dentin matrix protein 1; PHEX, phosphate-regulating gene homologous to endopeptidase on X chromosome.

The added P level affected (*p* ≤ 0.0005) *PHEX, DMP1*, and *MEPE* mRNA expressions but did not affect (*p* = 0.360) *FGF23* mRNA expression in primary cultured tibial osteoblasts of broiler chicks (Figure 3). The *DMP1* and *PHEX* mRNA expressions increased (*p* ≤ 0.001) at 1.0 mmol/L of added P, followed by a decrease (*p* ≤ 0.038) at 1.5 mmol/L of added P and another increase (*p* ≤ 0.009) at 2.0 mmol/L of added P (Figures 3C,D). The *MEPE* mRNA expression increased linearly (*p* = 0.0002) and quadratically (*p* = 0.037) as added P levels increased (Figure 3B). The added P level affected MEPE protein expression but did not affect (*p* > 0.156) FGF23, PHEX, and DMP1 protein expressions (Figure 4). The protein expression of MEPE increased quadratically (*p* = 0.005) and reached a peak at 1.0 mmol/L of added P (Figure 4B).

### MRNA and Protein Expressions of BMP/MAPK Pathway

**Figure 5:**
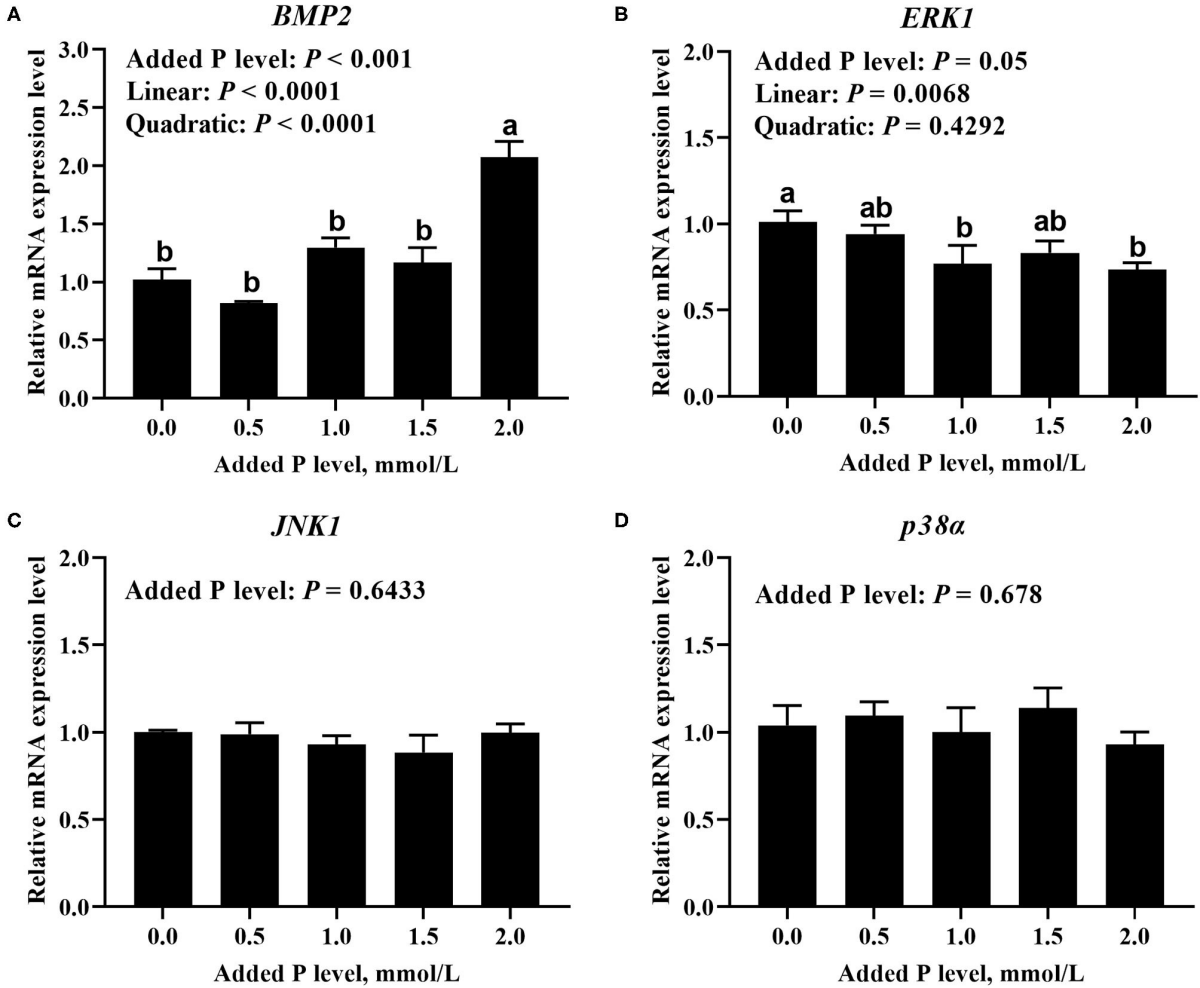
Effect of added P level on the mRNA expression levels of *BMP2* **(A)**, *ERK1* **(B)**, *JNK1* **(C)**, and *p38*α **(D)** in primary cultured tibial osteoblasts of broiler chicks. Each value was mean ± SE, n = 6. Means without a common letter differ, *p* ≤ 0.05. *BMP2*, bone morphogenetic protein 2; *ERK1*, extracellular signal-regulated kinase 1; *JNK1*, c-Jun N-terminal kinase 1; *p38*α, p38 mitogen-activated protein kinase alpha.

**Figure 6:**
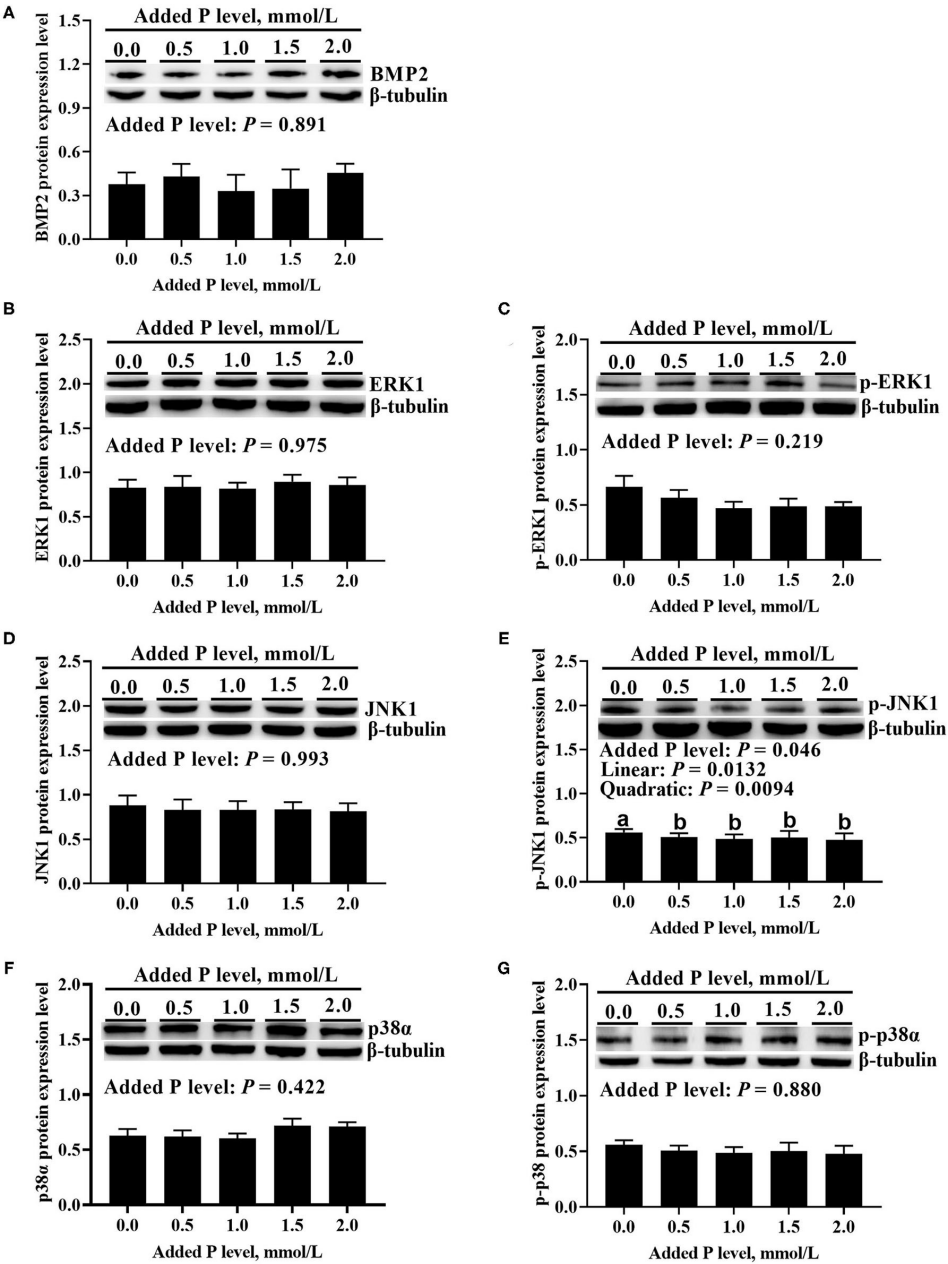
Effect of added P level on BMP2 protein expression level **(A)**, total and phosphorylated protein expression levels of ERK1 **(B,C)**, JNK1 **(D,E)**, and p38α **(F,G)** in primary cultured tibial osteoblasts of broiler chicks. Representative Western blots for these proteins were shown. Each value was mean ± SE, *n* = 6. Means without a common letter differ, *p* ≤ 0.05. BMP2, bone morphogenetic protein 2; ERK1, extracellular signal-regulated kinase 1; p-ERK1, phosphorylated extracellular signal-regulated kinase 1; JNK1, c-Jun N-terminal kinase 1; p-JNK1, phosphorylated c-Jun N-terminal kinase 1; p38α, p38 mitogen-activated protein kinase alpha; p-p38α, phosphorylated p38 mitogen-activated protein kinase alpha.

The added P level affected (*p* ≤ 0.05) *BMP2* and *ERK1* mRNA expressions but did not affect (*p* > 0.643) *JNK1* and *p38*α mRNA expressions in primary cultured tibial osteoblasts of broiler chicks (Figure 5). The *BMP2* mRNA increased linearly (*p* < 0.0001) and quadratically (*p* < 0.0001), whereas the *ERK1* mRNA expression decreased linearly (*p* < 0.007) as added P levels increased (Figures 5A,B). The added P level affected (*p* = 0.046) p-JNK1 protein expression but did not affect (*p* > 0.290) total and phosphorylated protein expression levels of other genes (Figure 6). The p-JNK1 protein expression decreased linearly (*p* = 0.013) and quadratically (*p* = 0.009) as added P levels increased (Figure 6E).

### Correlations Between Phosphorus Utilization Parameters and Local Bone-Derived Regulators or BMP/MAPK Pathway

The correlations between P utilization parameters (tibial osteoblastic P retention rate and number and area of mineralized nodules) and local bone-derived regulators or BMP/MAPK pathways are presented in Table 2. The tibial osteoblastic P retention rate was positively correlated (r = 0.452–0.564, *p* < 0.028) with *MEPE* and *BMP2* mRNA expressions; the number and area of mineralized nodules were positively correlated (r = 0.414–0.612, *p* < 0.03) with *PHEX, DMP1, MEPE*, and *BMP2* mRNA expressions but negatively correlated (r = −0.566 to 0.414, *p* < 0.04) with *ERK1* mRNA expression and p-JNK1 protein expression; all of the P utilization parameters were not correlated (*p* > 0.05) with MEPE protein expression.

**Table 2:** The correlation coefficients between P utilization parameters and local bone-derived regulators or BMP/MAPK pathway in primary cultured tibial osteoblasts of broiler chicks^a^.

**Items**	***MEPE* mRNA**	***DMP1* mRNA**	***PHEX* mRNA**	***BMP2* mRNA**	***ERK1* mRNA**	**p-JNK1**
Tibial osteoblastic *P* retention rate	0.452^*^	0.359	0.359	0.564^**^	−0.301	−0.317
Number of mineralized nodules	0.429^*^	0.524^**^	0.541^**^	0.554^**^	−0.502^**^	−0.566^**^
Area of mineralized nodules	0.414^*^	0.532^**^	0.559^**^	0.612^**^	−0.414^**^	−0.486^*^

*^a^Data represent the correlation coefficients between P utilization parameters and local bone-derived regulators or BMP/MAPK pathway in primary cultured tibial osteoblasts of broiler chicks, n = 30*.

* *p < 0.05,*

** *p < 0.01*.

*MEPE, matrix extracellular phosphoglycoprotein; DMP1, dentin matrix protein 1; PHEX, phosphate-regulating gene homologous to endopeptidase on X chromosome; BMP2, bone morphogenetic protein 2; ERK1, extracellular signal-regulated kinase 1; p-JNK1, phosphorylated c-Jun N-terminal kinase 1*.

## Discussion

In order to improve the bone P utilization efficiency of broilers and reduce its excretion to the environment, it is necessary to investigate the underlying regulatory mechanisms of the bone P utilization first. The hypothesis that P utilization in an osteoblastic model *in vitro* might be partly regulated by local bone-derived regulators and BMP/MAPK pathway has been supported by the results of the present study. The present study indicated that as added P levels increased, tibial osteoblastic P retention rate, number and area of mineralized nodules, the osteoblastic expressions of *DMP1* mRNA, *PHEX* mRNA, MEPE mRNA, and protein, *BMP2* mRNA, *ERK1* mRNA, and p-JNK increased or decreased linearly or quadratically. Correlation analyses showed that the above local bone-derived regulators and BMP/MAPK pathway genes were positively or negatively associated with the above P utilization parameters, suggesting that they might partly regulate the P utilization in primary cultured tibial osteoblasts of broiler chicks and thus might be potential targets for improving the bone P utilization of broilers. These findings provide a novel insight into the regulation of the P utilization and its mechanisms in the bone of broilers and will contribute to develop feasible strategies to improve the bone P utilization efficiency of broilers so as to decrease its excretion.

Dietary P supply plays a critical role in bone development and mineralization of broiler chickens (2, 3, 5, 6), since a high proportion of P was deposited in the bone as the component of hydroxyapatite (11). Moreover, recently emerging evidence indicated that the body P utilization efficiency in birds was shown to have strong phenotypic correlations with bone development and bone P utilization parameters like the tibial P retention efficiency (33). Therefore, bone P retention and bone mineralization-associated parameters can be considered good indicators for P utilization in broilers (34). Likewise, *in vitro*, the hydroxyapatite has been identified as the major component in the mineralized nodules (35). Thus, number and area of mineralized nodules may be considered as the suitable indices for evaluating P utilization *in vitro*. A series of studies showed that bone ash, P retention, and bone mineral content and density were affected by dietary P level, and increased linearly or quadraticly as dietary P levels increased (2, 3, 5, 36). Consistent with the *in vivo* findings, the results of the present study indicated that tibial osteoblastic P retention rate and number and area of mineralized nodules increased linearly and quadraticly as added P levels increased. However, the underlying regulatory mechanisms of P utilization are not well known.

Local bone-derived regulators, such as FGF23, MEPE, DMP1, and PHEX, have emerged as important regulators of P homeostasis and bone mineralization (37). Of these, DMP1 and PHEX are osteogenic marker genes, whose mutations result in hypophosphatemia, low bone mineral density, and elevated FGF23 production (38, 39). Moreover, DMP1 and FGF23 have been identified as P-responsive genes. Nishino et al. (17) reported that elevated extracellular P facilitated osteoblastic differentiation by upregulating DMP1 expression. An elevation of dietary P level induced tibia FGF23 expression in broilers (40). The MEPE is a component of bone matrix, and its overexpression in mice displayed growth and bone mineralization defects characterized by low bone mass, low bone density, etc. (41). In the present study, osteoblastic *DMP1, PHEX* mRNA, MEPE mRNA, and protein expressions increased as added P level increased, while osteoblastic *FGF23* mRNA expression and protein expressions of DMP1, PHEX, and FGF23 were not affected by added P level. In an *in vivo* study of broilers, Cao et al. (5) found that tibia *DMP1* mRNA expression increased as dietary P level increased, while FGF23 and PHEX protein expressions were not affected by dietary P level, which is in agreement with the above *in vitro* results of the present study. However, Cao et al. (5) also reported that *FGF23* mRNA expression increased, and *PHEX* mRNA expression and DMP1 protein expression decreased as dietary P level increased, while MEPE mRNA and protein expressions were not affected by dietary P level, which is not consistent with the above *in vitro* results of the present study. Notably, the *DMP1* and *PHEX* mRNA expressions increased and reached a peak at 1.0 mmol/L of added P level, indicating an induced role of elevated extracellular P in DMP1 and PHEX expressions (17, 42). But interestingly, they decreased at 1.5 mmol/L of added P, followed by another increase at 2.0 mmol/L of added P, and the exact reasons are unclear and need to be further studied. Moreover, correlation analyses in the current study showed that the osteoblastic P utilization parameters were positively associated with *MEPE, DMP1*, and *PHEX* mRNA expressions. In broilers (5), *DMP1* mRNA expression had positive correlation with bone P retention parameters, which is consistent with the above results of the present study; however, bone P retention parameters were positively associated with *FGF23* mRNA expression but negatively associated with *PHEX* mRNA expression, which is not consistent with the above results of the present study. No other similar studies have been reported before. The above disparities between *in vivo* and *in vitro* findings might be due to the effects of other *in vivo* factors except for P, such as the feed intake and hormones. However, *in vitro*, P was a single impacting factor, and thus, the *in vitro* results might better reflect the specific and direct effect of P itself on the above local bone-derived regulators. The above results from the present study suggested that the osteoblastic P utilization might be partly regulated by these local bone-derived regulators DMP1, MEPE, and PHEX in primary cultured tibial osteoblasts of broiler chicks. However, further studies are needed to confirm whether and how DMP1, MEPE, and PHEX are involved in regulating the osteoblastic P utilization of broilers.

The BMP/MAPK pathway plays a key role in regulating osteoblastic differentiation and mineralization (19, 22, 23). The BMP is a multifunctional acidic polypeptide, which is predominantly synthesized and secreted by osteoblasts (43). Within the BMP family, BMP2 is a potent inducer of bone formation through its stimulation of osteoblast differentiation (44, 45). Sun et al. (21) found that overexpression of BMP2 in bone mesenchymal stem cells not only induced ALP activity but also promoted the formation of mineralization nodules. Moreover, BMP2 has been reported to exert its osteogenic function *via* MAPK pathway. Bokui et al. (46) reported that BMP2 activated the p38, ERK1/2, and JNK1/2 to promote the expression of Runx2, an osteogenic-specific transcription factor. Earlier studies also showed that p38, ERK, and JNK were essential for BMP2-induced osteoblastic differentiation and mineralization (23, 47). In the present study, osteoblastic *BMP2* mRNA expression increased, and the expressions of ERK1 mRNA and p-JNK decreased as added P level increased, while the expressions of *p38*α and *JNK1* mRNA, BMP2 protein expression, the total or phosphorylated protein expressions of ERK1 and p38α, and the total protein expression of JNK1 were not affected by added P level. In an *in vivo* study of broilers, Liao et al. (24) found that tibia *ERK1* mRNA expression decreased as dietary P level increased, while total protein expressions of ERK1 and JNK1, and total and phosphorylated protein expressions of p38α were not affected by dietary P level, which is in agreement with the above *in vitro* results of the present study. However, Liao et al. (24) also observed that the expressions of *p38*α mRNA, BMP2, and p-ERK proteins decreased as dietary P level increased, while the expressions of *BMP2* mRNA and p-JNK protein were not affected by dietary P level, which is not consistent with the above *in vitro* results of the present study. Furthermore, correlation analyses in the present study showed that osteoblastic P utilization parameters were positively correlated with *BMP2* mRNA expression but negatively correlated with *ERK1* mRNA expression and p-JNK1 protein expression. In broilers (24), bone P retention parameters were negatively correlated with *ERK1* mRNA expression, which is consistent with the above results of the present study, but also negatively correlated with the expressions of *JNK1* mRNA, BMP2, and p-ERK protein, which is not consistent with the above results of the present study. No other similar studies have been available so far. The above differences between *in vivo* and *in vitro* findings might be caused by other *in vivo* factors except for P, such as the feed intake and hormones. Therefore, the results from the above *in vivo* and *in vitro* studies have pointed out a possible role of BMP/MAPK pathway in regulating the bone P utilization in broilers.

## Conclusions

The results from the present study indicated that P utilization parameters (tibial osteoblastic P retention rate and number and area of mineralized nodules) had positive correlations with *MEPE, DMP1, PHEX*, and *BMP2* mRNA, but negative correlations with *ERK1* mRNA and phosphorylated JNK1 in primary cultured osteoblasts of broiler chicks, and thus, they might be partly regulated by these local bone-derived regulators and BMP/MAPK pathway. Further studies are necessary to verify the roles of these local bone-derived regulators and BMP/MAPK pathway in regulating the osteoblastic P utilization of broilers.

## Data Availability Statement

The original contributions presented in the study are included in the article/supplementary material, further inquiries can be directed to the corresponding author.

## Ethics Statement

All experimental procedures were approved by the Animal Management Committee (in charge of animal welfare issue) of the Institute of Animal Science, Chinese Academy of Agricultural Sciences (IAS-CAAS, Beijing, China) and performed in accordance with the guidelines. Ethical approval on animal survival was given by the animal ethics committee of IAS-CAAS.

## Author Contributions

TL: data curation and writing—original draft preparation. SC, XLi, and LZ: investigation. YS: formal analysis. LL: methodology. ZL: resources. XLu: supervision and writing—review and editing. All authors contributed to the article and approved the submitted version.

## Funding

This work was supported by the Key Program of the National Natural Science Foundation of China (project no. 31630073; Beijing, China), Initiation Funds of Yangzhou University for Distinguished Scientists (Yangzhou, China), the National Key R&D Program of China (project no. 2017YFD0502200; Beijing, China), the China Agriculture Research System of MOF and MARA (project no. CARS-41; Beijing, China), and the Agricultural Science and Technology Innovation Program (project no. ASTIP-IAS09; Beijing, China).

## Conflict of Interest

The authors declare that the research was conducted in the absence of any commercial or financial relationships that could be construed as a potential conflict of interest.

## Publisher's Note

All claims expressed in this article are solely those of the authors and do not necessarily represent those of their affiliated organizations, or those of the publisher, the editors and the reviewers. Any product that may be evaluated in this article, or claim that may be made by its manufacturer, is not guaranteed or endorsed by the publisher.
